# The Role of DNMT and HDACs in the Fetal Programming of Hypertension by Glucocorticoids

**DOI:** 10.1155/2020/5751768

**Published:** 2020-03-28

**Authors:** J. Lamothe, S. Khurana, S. Tharmalingam, C. Williamson, C. J. Byrne, N. Khaper, S. Mercier, T. C. Tai

**Affiliations:** ^1^Biomolecular Sciences, Laurentian University, Sudbury, ON, Canada; ^2^Medical Science Division, Northern Ontario School of Medicine Sudbury, ON, Canada; ^3^Biology, Laurentian University, Sudbury, ON, Canada; ^4^Medical Science Division, Northern Ontario School of Medicine Thunder Bay, ON, Canada; ^5^School of Human Kinetics, Laurentian University, Sudbury, ON, Canada

## Abstract

The causes of hypertension are complex and involve both genetic and environmental factors. Environment changes during fetal development have been linked to adult diseases including hypertension. Studies show that timed in utero exposure to the synthetic glucocorticoid (GC) dexamethasone (Dex) results in the development of hypertension in adult rats. Evidence suggests that *in utero* stress can alter patterns of gene expression, possibly a result of alterations in the topology of the genome by epigenetic markers such as DNA methyltransferases (DNMTs) and histone deacetylases (HDACs). The objective of this study was to determine the effects of epigenetic regulators in the fetal programming and the development of adult hypertension. Specifically, this research examined the effects of the HDAC inhibitor valproic acid (VPA) and the DNMT inhibitor 5-aza-2′-deoxycytidine (5aza2DC) on blood pressure (BP) and gene expression in prenatal Dex-programmed rats. Data suggest that both VPA and 5aza2DC attenuated the Dex-mediated development of hypertension and restored BP to control levels. Epigenetic DNMT inhibition (DNMTi) or HDAC inhibition (HDACi) also successfully attenuated elevations in the majority of altered catecholamine (CA) enzyme expression, phenylethanolamine *N*-methyltransferase (PNMT) protein, and elevated epinephrine (Epi) levels in males. Although females responded to HDACi similar to males, DNMTi drove increased glucocorticoid receptor (GR) and PNMT expression and elevations in circulating Epi in females despite showing normotensive BP.

## 1. Introduction

Despite many advances in hypertension research, factors contributing to the development of the disease continue to emerge. Increasing significance has been placed on the fetal environment in the pathophysiology of the disease. Of particular interest is the role of GCs in fetal development and programming [1]. GCs can stimulate tissue maturation and fetal development; however, in times of stress, excess production of maternal GCs can negatively impact the fetus, promoting premature tissue development and programing for disease [1]. As outlined in our previous paper, understanding how these changes in the fetal environment affect adult gene expression has been the key to unravelling the mechanisms involved in the fetal programming of hypertension [2]. Previous research has highlighted a role for ROS in programming; however, epigenetic modifications such as DNA methylation and histone acetylation are suspected to propagate these fetal insults to postnatal health. Increased fetal GC exposure may mediate programming through alteration of gene DNA methylation status; studies show increased global DNA methylation status in specific tissues, including the adrenal glands following betamethasone administration in guinea pigs [3]. The adrenal gland is the downstream regulator of the HPA axis, responsible for the production of CAs including Epi and norepinephrine (NE). Many genes suspected to be implicated in the development of hypertension are regulated via promoter methylation by DNMTs and are present in the adrenal gland [4, 5]. Given that GCs mediate large changes in the adrenal methylation status, it is likely a result of altered DNMT abundance or activity. DNMTi has been shown to prevent NE-induced cardiac hypertrophy in rats [6], which may provide evidence for DNMT propagating fetal insults via GCs to gene expression changes in adulthood. DNMTs have also been implicated in a pulmonary model of hypertension, where DNMTi was shown to restore SOD2 expression and attenuate disease [7]. DNMTs have also been shown to mediate changes in mitochondrial function in times of stress, leading to altered ROS production [8]. As a result, not only are DNMTs implicated in hypertensive gene expression changes, DNMTs may also mediate ROS through changes in antioxidant enzyme expression patterns and mitochondrial ROS production.

HDACs are also involved in the development of hypertension. HDACi via VPA has been shown to be effective in attenuating inflammation, hypertrophic, and hypertensive responses in a spontaneously hypertensive rat (SHR) model of hypertension [9]. Interestingly, HDACi has proven to be effective in remediating hypertension in a rat model of Cushing's syndrome, characterized by excess GC cortisol [10]. HDACs have also been linked to pulmonary [11], high-fat diet-induced [12], and angiotensin II-induced [13] hypertension, and HDACi has been proven effective in treatment of hypertension. HDACi via trichostatin A administration has also been proven effective in attenuating neonatal Dex programming [14]. However, the role of HDACs in GC-mediated fetal programming of the HPA axis remains unknown. Similar to DNMTs, there is much overlap between HDACs and ROS, as HDACs have been shown to promote NADPH oxidase (Nox) expression and increase ROS production [15].

This study aims to investigate the role of DNMT or HDAC inhibition in GC-mediated fetal programming of hypertension. Specifically, the DNMT inhibitor 5aza2DC and the HDAC inhibitor VPA will be employed in GC-programmed adults, to determine if epigenetic inhibition can reverse hypertensive programming of CA biosynthesis and overall phenotype.

## 2. Methods

### 2.1. Animals

Male (*n* = 6) and female (*n* = 18) Wistar-Kyoto rats were purchased from Charles River Laboratories (Montreal, QC, Canada) at 6 weeks of age. Upon arrival, rats were housed in groups of 2-3 and allowed to acclimate to their new environment until 10 weeks of age. All animals were provided with food and water *ad libitum.* Procedures were followed as per Canadian Council on Animal Care guidelines and were approved by Laurentian University Animal Care Committee.

### 2.2. Breeding and Experimental Design

At 10 weeks of age, a male was housed with a group of three females until vaginal plugs were observed (gestational day 0 (GD 0)). Pregnant females were separated and housed individually. They then received a subcutaneous injection of Dex (100 *μ*g/kg/day in 4% ethanol/0.9% saline) or saline injection (4% ethanol/0.9% saline) from GD -15-21 [2]. Following birth, offspring were weaned at 3 weeks of age and split into male and female groups.

### 2.3. Blood Pressure Measurements

The CODA-8 (Kent Scientific) noninvasive volume pressure recording tail-cuff BP system was employed to record BP measurements as performed previously [2]. Offspring were acclimated to the machine beginning at week 3 and measurements began at week 4 and ran until sacrifice at the end of week 14. Animals were also acclimated to the machine for 10 minutes prior to measurements. A total of 25 readings were taken per day over the course of 25 minutes, and three days of each week were recorded to form an average measurement per week. Measurements were recorded prior to any husbandry duties and within the hours of 9 am to 6 pm to avoid diurnal variation in BP.

### 2.4. Injections

**Figure 1:**
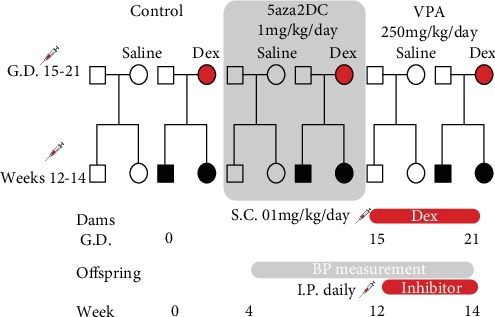
Schematic of fetal programming and epigenetic inhibitor administration. Pregnant WKY dams received 100 *μ*g/kg/day dexamethasone S.C. from gestational days 15-21. Pups exposed to Dex *in utero* are shown in black. Weekly BP measurements were taken from weeks 4 to 14. Injections with the DNMT inhibitor 5aza2DC or VPA began at week 12 until sacrifice at the end of week 14. Adrenal glands were collected for protein and gene expression analysis, and plasma was collected for catecholamine analysis.

In this study, offspring from saline- and Dex-injected dams received injections of VPA or 5Aza2DC and were categorized into 6 groups (Saline-Control, Dex-Control, Saline-5aza2DC, Dex-5aza2DC, Saline-VPA, and Dex-VPA); *n* = 6 animals per sex per group. Beginning at week 12 (day 78), once Dex-exposed animals displayed elevated BP relative to controls, animals received daily injections of saline (0.9%), 5aza2DC (1 mg/kg/day; LC Labs), or VPA (250 mg/kg/day; Cayman Labs) I.P. (Figure 1) for a total of 20 injections by the end of week 14.

### 2.5. Tissue Collection and Extraction

Animals were sacrificed upon the completion of week 14 epigenetic inhibitor injections. Animals were anesthetized via an injection of 75 mg/kg Ketalean (Ketalean; Bimeda, Cambridge, ON) and 5 mg/kg xylazine (Rompun; Bayer, Etobicoke, ON) I.P. and sacrificed using decapitation [16]. Following decapitation, trunk blood was collected into EDTA-coated (10.8 mg) Vacutainer blood collection vials (Becton Dickinson, Franklin Lakes, NJ, USA), and tissues including adrenal glands were harvested and flash frozen on dry ice for future analysis [2].

### 2.6. Adrenal mRNA Expression

Adrenals were homogenized using stainless steel beads and run in the TissueLyser (Qiagen) with TRIzol Reagent (Sigma-Aldrich) [2]. Following RNA extraction, RNA pellets were resuspended in DEPC-treated nuclease-free water. Quantification of RNA samples was assessed via a Nanodrop 1000 spectrophotometer (260 nm). Of the RNA samples, 2 *μ*g were then treated with DNase I (Sigma) and converted to DNA using M-MLV reverse transcriptase (Promega) [2]. Gene expression was assessed using qPCR with Bioline SensiFast Sybr Lo-Rox mix (FroggaBio) and run on a Chromo4 qPCR system (BioRad).  Table 1 highlights the primers used in qPCR analysis. A custom RT^2^ profiler array (Qiagen) was employed to assess the expression of epigenetic regulations and additional antioxidant pathway targets. Total volume of 15 *μ*L for qPCR reactions with 7.5 ng input cDNA. Primers for PNMT, TH, DBH, GR, EGR-1 SP1, RPL29, and B-actin were obtained from Sigma-Aldrich. Fold change was determined using the Ct value for each sample via the Pfaffl method ratio = (*E*_target_)^ΔCTtarget(cotrol − sample)^/(*E*_ref_)^ΔCTref(cotrol − sample)^ [17]. qPCR experiments were run in duplicate, with a biological replicate of *N* = 6 animals unless stated otherwise.

### 2.7. Western Blot

The All-Prep kit from Qiagen was employed to isolate protein from half of the remaining adrenal gland. Again, homogenization was performed with the TissueLyser (Qiagen). The protein-solubilizing buffer was supplemented with DTT to a total concentration of 8 mg DTT/1 mL ALO buffer to optimize pellet solubilization. ALO (300 *μ*L) was then added to protein pellets, and samples were sonicated for 10 s at 100% amplitude (Sonic Dismembrator 500, Fisher Scientific). Following resuspension, samples were stored at -80°C until western blot analysis could be performed. A total of 5 *μ*L of each sample was used for western blot, and gels were run as performed previously [2]. Gels were transferred to nitrocellulose membranes. Blots probing for DBH were blocked with 2% BSA. Primary antibodies used include TH (Novus Biologicals), DBH (Abcam), PNMT (Abcam), SP1 (Santa-Cruz), GR (Abcam), and GAPDH (Abcam). Based on primary antibody origin, secondaries conjugated to HRP-IgG were used. Blots were incubated for 2 minutes with ECL and exposed to a film for band visualization as described by Haan and Behrmann [18]. Quantification was performed using ImageJ (U.S. National Institutes of Health, Bethesda, MD, USA), and gels were normalized to GAPDH control. PAH and EGR1 were not quantified as antibodies suitable for western blot were not readily available.

### 2.8. Corticosterone and Catecholamine Levels

Following short-term storage on ice, plasma was separated from blood samples via centrifugation at 1500 g for 20 min and stored at -80°C [2]. Plasma (50 *μ*L) was then run through the 2-CAT ELISA from LDN (Rocky Mountain Diagnostic, Colorado Springs, CO, USA) to quantify plasma Epi and NE levels as per manufacturer's instructions. The parameter corticosterone ELISA from R&D systems (Minneapolis, MN, USA) was employed to assess plasma corticosterone levels.

### 2.9. Quantification and Statistical Analysis

Statistical computation was performed via the use of GraphPad PRISM software (La Jolla, CA, USA). Data is represented as mean ± SEM, and significance is indicated as ^∗^*P* ≤ 0.05, ^∗∗^*P* ≤ 0.01, ^∗∗∗^*P* ≤ 0.001, ^∗∗∗∗^*P* ≤ 0.0001. ∗ is relative to Control-Saline group, and † is relative to the Dex-Control group.

## 3. Results

**Figure 2:**
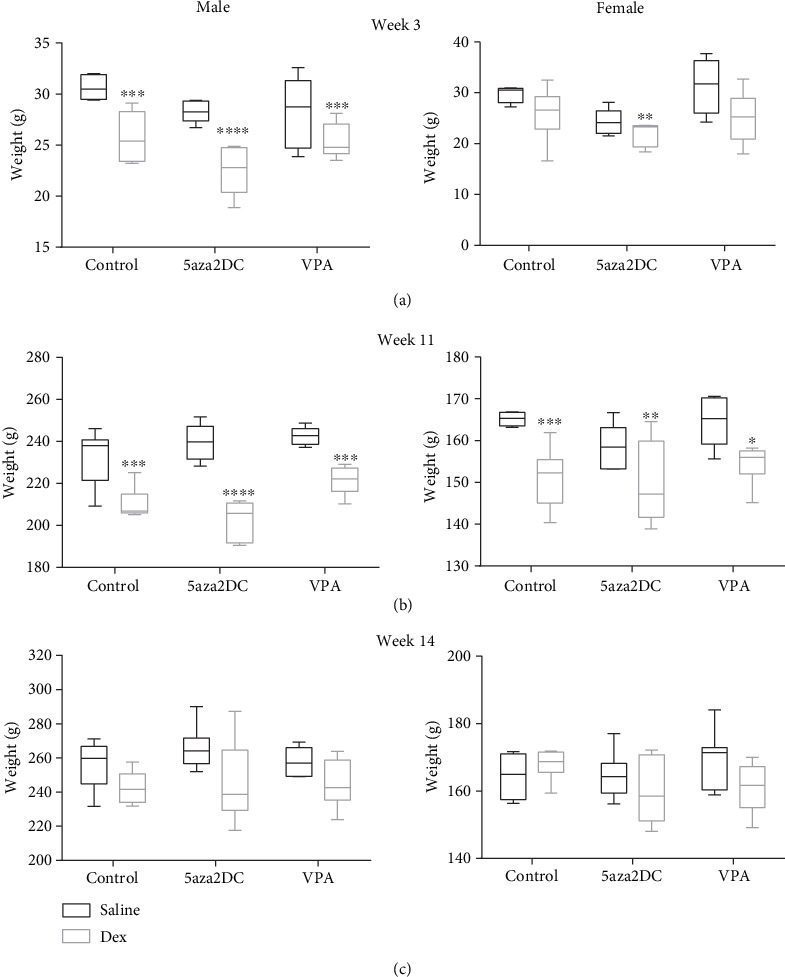
Animal body weights. Male and female body weights of Dex-programmed offspring at (a) week 3 after birth, (b) before administering drugs week 11, (c) and after receiving 5aza2DC or VPA via daily injections (I.P.) from weeks 12 to 14 at week 14. *N* = 6. Two-way ANOVA (Fisher LSD test): statistical significance is represented as ^∗^*P* = 0.05, ^∗∗^*P* = 0.01, ^∗∗∗^*P* = 0.001, and ^∗∗∗∗^*P* = 0.0001. The symbol (^∗^) indicates significance relative to Saline-Control. Plot wings represent min and max values within each group. *N* = 6 per group.

**Figure 3:**
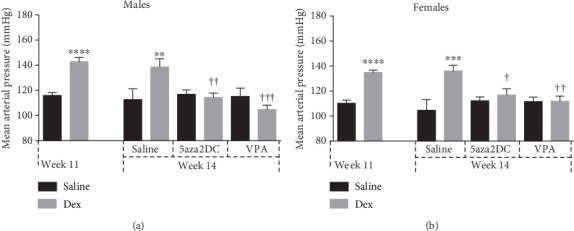
Mean arterial pressure of Dex-programmed offspring exposed to epigenetic inhibitors in adulthood. Mean arterial pressure of Dex-programmed offspring given 5aza2DC or VPA in (a) males or (b) females via daily injections (I.P.) from weeks 12 to 14. Measured using the CODA8 BP monitor from Kent Scientific. *N* = 6. Two-way ANOVA (Fisher LSD test): statistical significance is represented as ^∗/†^*P* ≤ 0.05, ^∗∗/††^*P* ≤ 0.01, ^∗∗∗/†††^*P* ≤ 0.001, and ^∗∗∗∗/††††^*P* ≤ 0.0001. Saline-Control and Dex-Control are shown here for reference, and results have been described previously. ∗ indicates significance relative to Saline-Control, and † indicates significance relative to Dex-Control. Data are presented as mean ± SEM. *N* = 6 per group.

As expected, Dex-programmed males (25.3 g) showed reduced body weight shortly after birth compared to Saline-Control (30.3 g) at weeks 3 of age, with females displaying similar trends (Figure 2(a)) [16]. Dex-programmed males and females continue to display reduced body weight compared to controls at week 11, until week 14 when significance is lost, yet the trend remains the same (Figures 2(b)–2(c)). By week 11, prenatally Dex-exposed male and female offspring display significantly increased BP compared to Saline-Control (Figures 3(a) and 3(b)). The epigenetic inhibitors 5aza2DC and VPA were effective in attenuating elevated BP induced by prenatal Dex exposure in adult offspring for both sexes (Figures 3(a) and 3(b)). 5aza2DC administration in Dex-programmed male offspring decreased mean arterial pressure (MAP) from 155 mmHg at week 11 to 117 mmHg by week 14 compared to Dex-Control animals which displayed a MAP of 140 mmHg by the end of week 14 (Figure 3(a)). 5aza2DC alone did not affect BP compared to Saline-Control (Figure 3(a)). Similarly, females administered 5aza2DC in the Dex-programmed group decreased MAP from 131 mmHg at week 11 to 117 mmHg at week 14 compared to Dex-Control animals which were at 134 mmHg at the end of week 14 (Figure 3(b)).

VPA administration in programmed male offspring decreased MAP from 140 mmHg to 109 mmHg by week 14, while Dex-Control animals maintained elevated MAP of 140 mmHg by week 14 (Figure 3(a)). Similarly, administration of VPA in Dex-exposed females decreased MAP from 133 mmHg to 112 mmHg by week 14 compared to Dex-Control females which displayed a MAP of 134 mmHg at week 14 (Figure 3(b)).

### 3.1. Gene Expression Analysis

**Figure 4:**
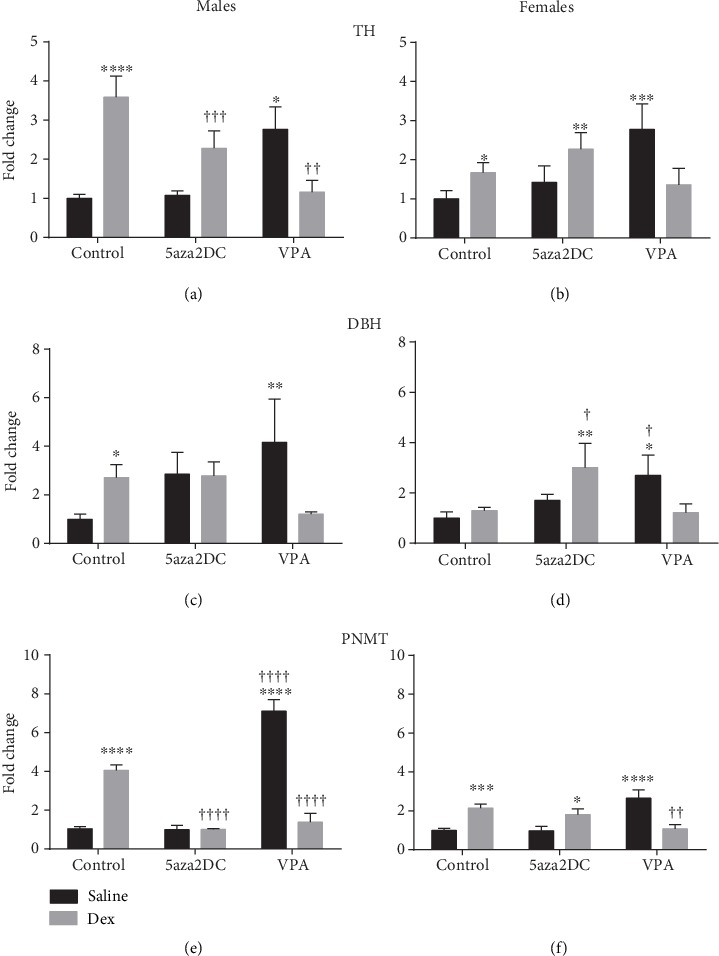
Relative mRNA expression of catecholamine biosynthetic enzyme expression (2^-*ΔΔ*Ct^). qPCR gene expression of catecholamine biosynthetic enzymes including (a, b) tyrosine hydroxylase (TH), (c, d) dopamine beta-hydroxylase (DBH), and (e, f) phenylethanolamine *N*-methyltransferase (PNMT) for males and females. Two-way ANOVA (Fisher LSD test): statistical significance is represented as ^∗/†^*P* ≤ 0.05, ^∗∗/††^*P* ≤ 0.01, ^∗∗∗/†††^*P* ≤ 0.001, and ^∗∗∗∗/††††^*P* ≤ 0.0001. Saline-Control and Dex-Control are shown here for reference, and results have been described previously. Data are presented as mean ± SEM. ∗ indicates significance relative to Saline-Control, and † indicates significance relative to (c). *N* = 4‐6 per group.

**Figure 5:**
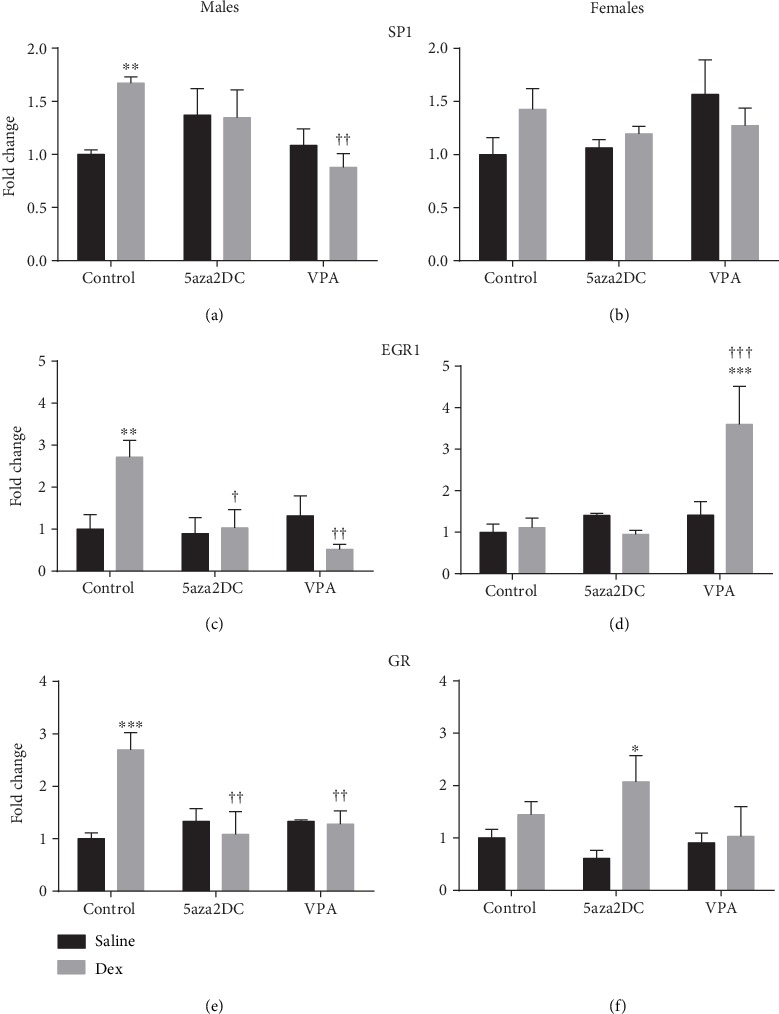
Relative mRNA expression of catecholamine biosynthetic enzyme transcription factor expression (2^-*ΔΔ*Ct^). qPCR gene expression of catecholamine biosynthetic enzymes transcription factor including (a, b) specificity protein 1 (SP1), (c, d) early growth response 1 (EGR1), and (e, f) glucocorticoid receptor (GR) for males and females. Two-way ANOVA (Fisher LSD test): statistical significance is represented as ^∗/†^*P* ≤ 0.05, ^∗∗/††^*P* ≤ 0.01, and ^∗∗∗/†††^*P* ≤ 0.001. Saline-Control and Dex-Control are shown here for reference, and results have been described previously. Data are presented as mean ± SEM. ∗ indicates significance relative to Saline-Control, and † indicates significance relative to Dex-Control. *N* = 3‐6 per group.

The expression of CA biosynthetic enzymes and related regulatory transcription factors are shown in Figures 4(a)–4(f) and 5(a)–5(f), respectively. Prenatal Dex exposure has been shown to increase the expression of CA biosynthetic enzymes including TH, DBH, and PNMT, particularly in males [2]. Administration of the DNMT inhibitor 5aza2DC was effective in attenuating some of the increased CA enzymes due to Dex exposure for males only (Figures 4(a), 4(c), and 4(e)). Male offspring within the Dex-5aza2DC group displayed a reduction in the expression of TH (2.3-fold) and PNMT (1.0-fold), compared to Dex-Control (3.6-fold and 4.1-fold, respectively) (Figures 4(a) and 4(e)). DBH remained elevated similar to Dex-Control levels. Interestingly, 5aza2DC affected female offspring differently than males, as Dex-exposed females maintained elevated expression of TH (2.2-fold), PNMT (1.8-fold), and DBH (3.0-fold) expression as compared to the untreated Dex-exposed females (Figures 4(b), 4(d), and 4(f)).

In contrast to DNMT inhibition, Dex-exposed animals given VPA shows a drastic reduction in CA enzyme expression for both sexes (Figures 4(a)–4(f)). In males, VPA reduced the expression of TH (1.2-fold), DBH (1.2-fold), and PNMT (1.4-fold) compared to Dex-Control groups (3.6-fold, 2.7-fold, and 4.1-fold, respectively) (Figures 4(a)–4(c)). Mirroring male offspring, female offspring in the Dex-VPA group shows a significant reduction in TH (2.2-fold) and PNMT (1.1-fold) compared to Dex-Control (3.2-fold and 2.1-fold, respectively) (Figures 4(a) and 4(e)). Interestingly, for male and female offspring, animals in the Saline-VPA group displayed significantly elevated levels of TH, DBH, and PNMT similar to that of Dex-Control animals (Figures 4(a)–4(f)).

Dex-exposed animals also display significantly increased gene expression levels of transcription factors SP1, EGR1, and GR predominantly in males as shown previously [2]. Both VPA and 5aza2DC were largely effective in reducing transcription factor expression levels in males. Dex-exposed females do show a trend towards increased SP1 and GR expression although not statistically significant (Figures 5(b) and 5(f)).

Treatment with 5aza2DC in prenatally Dex-exposed male offspring decreased the expression of SP1 (1.3-fold), EGR1 (1.0-fold), and GR (1.1-fold) to levels comparable to Saline-Control (Figures 5(a), 5(c), and 5(e)). In response to 5aza2DC, Dex-exposed female offspring did not show significant changes in transcription factor expression with the exception of elevated GR (2.1-fold) although the result is not significantly different then the Dex-Control group (1.4-fold) (Figure 5(f)).

VPA reduced the expression of SP1 (0.9-fold), EGR1 (0.5-fold), and GR (1.3-fold) in males as compared to Dex-Control levels and are comparable to Saline-Control (Figures 5(a), 5(c), and 5(e)). Interestingly, female offspring in the VPA-Dex (3.6-fold) group display elevated EGR1 compared to Saline-Control and Dex-Control (1.1-fold) groups (Figure 5(d)).

### 3.2. Protein Expression

**Figure 6:**
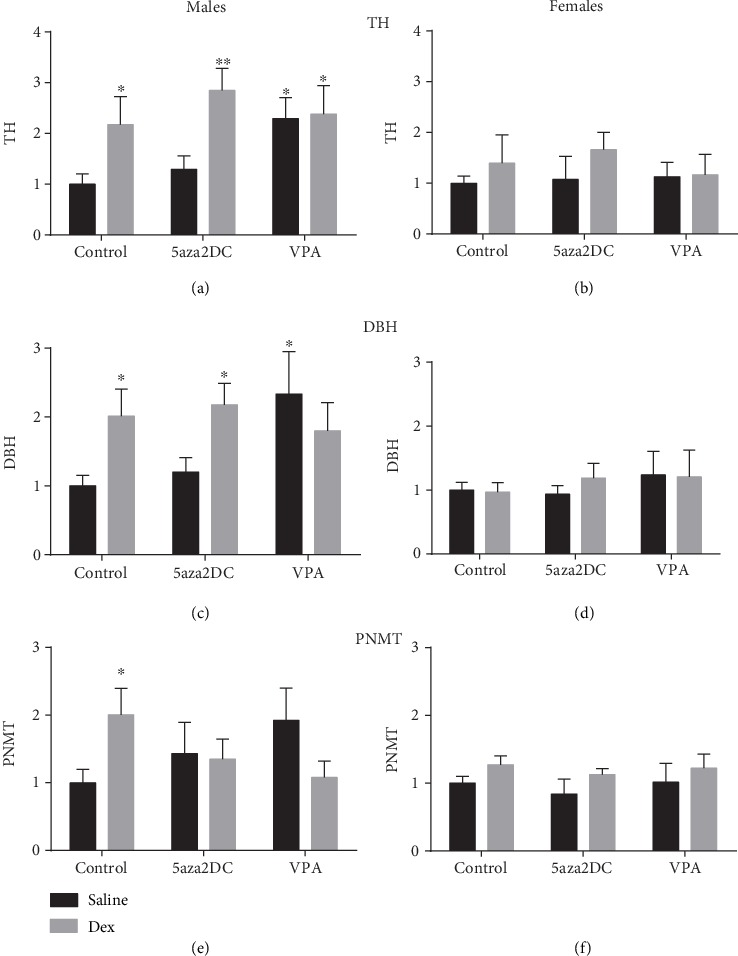
Quantification of catecholamine biosynthetic enzyme protein levels. Western blot analysis of (a, b) tyrosine hydroxylase (TH), (c, d) dopamine beta-hydroxylase (DBH), and (e, f) phenylethanolamine *N*-methyltransferase (PNMT) for males and females. Two-way ANOVA (Fisher LSD test): statistical significance is represented as ^∗^*P* = 0.05 and ^∗∗^*P* = 0.01. Saline-Control and Dex-Control are shown here for reference, and results have been described previously. Data are presented as mean ± SEM. ∗ indicates significance relative to Saline-Control, and † indicates significance relative to Dex-Control. *N* = 3‐6 per group.

Prenatal Dex exposure increased protein levels for CA biosynthetic enzymes TH (2.4-fold), DBH (2.0-fold), and PNMT (2.0-fold) in males, but not females (Figures 6(a), 6(c), and 6(e)). Control-Dex females do display an increasing trend in PNMT though not significant (Figure 6(f)). Administration of 5aza2DC did not attenuate TH (2.8-fold) or DBH (2.2-fold) protein levels; however, it was effective in reducing PNMT (1.3-fold) (Figures 6(a), 6(c), and 6(e)). 5aza2DC did not display a significant effect on protein levels in programmed and unprogrammed female offspring (Figures 6(b), 6(d), and 6(f)).

Similar to the DNMT inhibitor, VPA was largely effective at reducing PNMT protein levels in males (1.1-fold) compared to Dex-Control group (2.0-fold) (Figure 6(e)). Both TH (2.4-fold) and DBH (1.8-fold) remained elevated and were comparable to Dex-Controls (2.2-fold and 2.0-fold, respectively) (Figures 6(a) and 6(c)). Interestingly, elevated mRNA expression of CA genes in unprogrammed males administered VPA matched elevated protein levels: TH (2.3-fold) and DBH (2.3-fold), with PNMT displaying a similar trend although it did not reach statistical significance (Figures 6(a), 6(c), and 6(e)).

**Figure 7:**
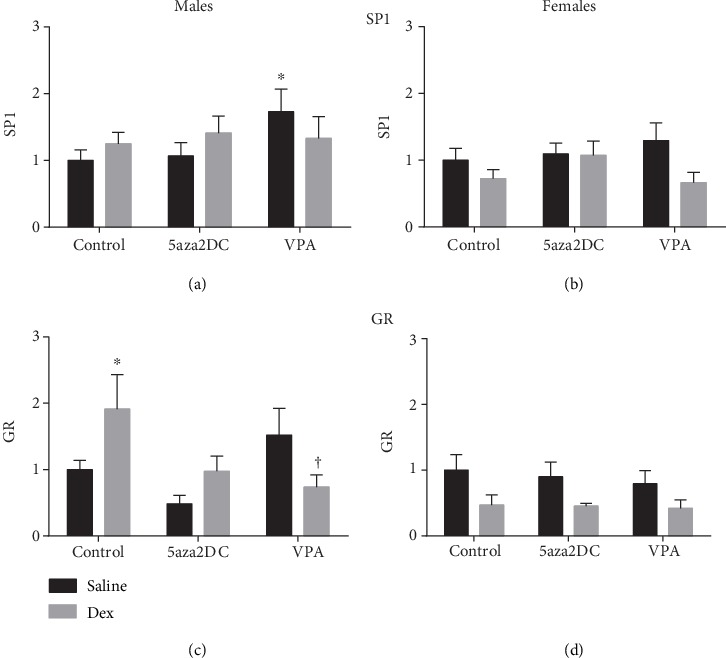
Quantification of catecholamine biosynthetic enzyme transcription factor protein levels from offspring adrenal gland. Western blot analysis of (a, b) specificity protein (SP1) and (c, d) glucocorticoid receptor (GR) for males and females. Two-way ANOVA (Fisher LSD test): statistical significance is represented as ^∗/†^*P* ≤ 0.05. Saline-Control and Dex-Control are shown here for reference, and results have been described previously. Data are presented as mean ± SEM. ∗ indicates significance relative to Saline-Control, and † indicates significance relative to Dex-Control. *N* = 3‐6 per group.

Contrary to gene expression results, prenatal Dex exposure did not program for increased protein levels of SP1 in adult males or females (Figures 7(a) and 7(b)). Males exposed to Dex do show a trend for elevated GR levels (1.7-fold) but not females (Figure 7(c)). Administration of the DNMT inhibitor 5aza2DC reduced GR protein levels (1.0-fold) in programmed males compared to Dex-Control (1.9-fold) (Figure 7(c)). 5aza2DC administration in females however did not significantly alter GR protein levels (Figure 7(d)).

VPA was effective in reducing Dex-driven increases in GR protein levels (0.7-fold) compared to Dex-Control (1.9-fold) in males (Figure 7(c)). VPA did not have an impact on prenatally Dex-exposed female offspring transcription factor expression (Figure 7(d)). Moreover, VPA-Saline male offspring shows significantly increased SP1 levels (1.7-fold) and a trend towards elevated levels of GR (Figures 7(a) and 7(c)) with females displaying a similar trend in SP1 expression (Figure 7(b)).

### 3.3. Plasma Catecholamines

**Figure 8:**
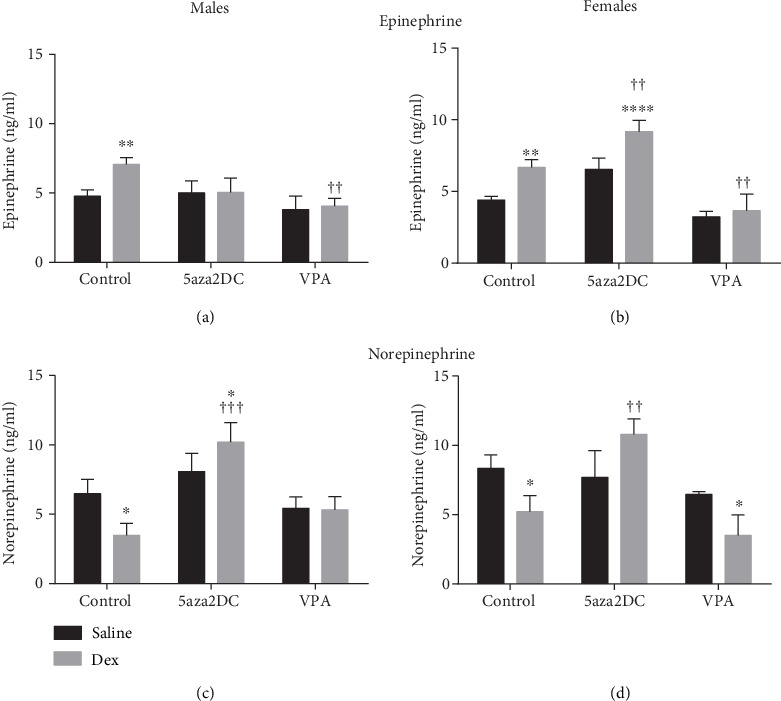
Plasma catecholamine levels. Results from plasma catecholamine ELISA for (a, b) epinephrine and (c, d) norepinephrine for males and females. Two-way ANOVA (Fisher LSD test): statistical significance is represented as ^∗/†^*P* ≤ 0.05, ^∗∗/††^*P* ≤ 0.01, ^∗∗∗/†††^*P* ≤ 0.001, and ^∗∗∗∗/††††^*P* ≤ 0.0001. Saline-Control and Dex-Control are shown here for reference, and results have been described previously. Data are presented as mean ± SEM. ∗ indicates significance relative to Saline-Control, and † indicates significance relative to Dex-Control. *N* = 4‐6 per group.

There was no significant change in corticosterone levels between treatment groups detectible with ELIS; however, changes in CA levels were detected following treatment with inhibitors of DNMT and HDAC. 5aza2DC was effective in reducing male Epi levels (from 7.1 ng/mL to 5.0 ng/mL) to that of Saline-Control (4.8 ng/mL) (Figure 8(a)); however, in females, 5aza2DC administration increased Epi levels (9.2 ng/mL) above Dex-Control animals (6.7 ng/mL) (Figure 8(b)). VPA was particularly effective in reducing Epi levels for both males (from 7.1 ng/mL to 4.1 ng/mL) and females (from 6.7 ng/mL to 3.7 ng/mL) as compared to Dex-Control groups (Figures 8(a) and 8(b)).

Interestingly, Dex-exposed offspring displayed a reduction in plasma NE levels: male (3.5 ng/mL) and female (5.2 ng/mL) compared to Saline-Control (6.5 ng/mL and 8.3 ng/mL, respectively) (Figures 8(c) and 8(d)). 5aza2DC increased NE levels above Saline-Control for males (10.2 ng/mL) and females (10.8 ng/mL), and as a result, the levels are significantly different than Dex-Control animals (Figures 8(c) and 8(d)).

VPA administration resulted in NE levels comparable to Saline-Control for unprogrammed and programmed male offspring (5.4 ng/mL and 5.3 ng/mL, respectively) (Figure 8(c)). Females however maintain a decrease in NE in the VPA-Dex group (3.5 ng/mL), significantly decreased from Saline-Control (8.3 ng/mL) and comparable to that of Dex-Control (5.2 ng/mL) (Figure 8(d)).

### 3.4. Additional Mechanisms in GC-Mediated Fetal Programming of Hypertension

**Figure 9:**
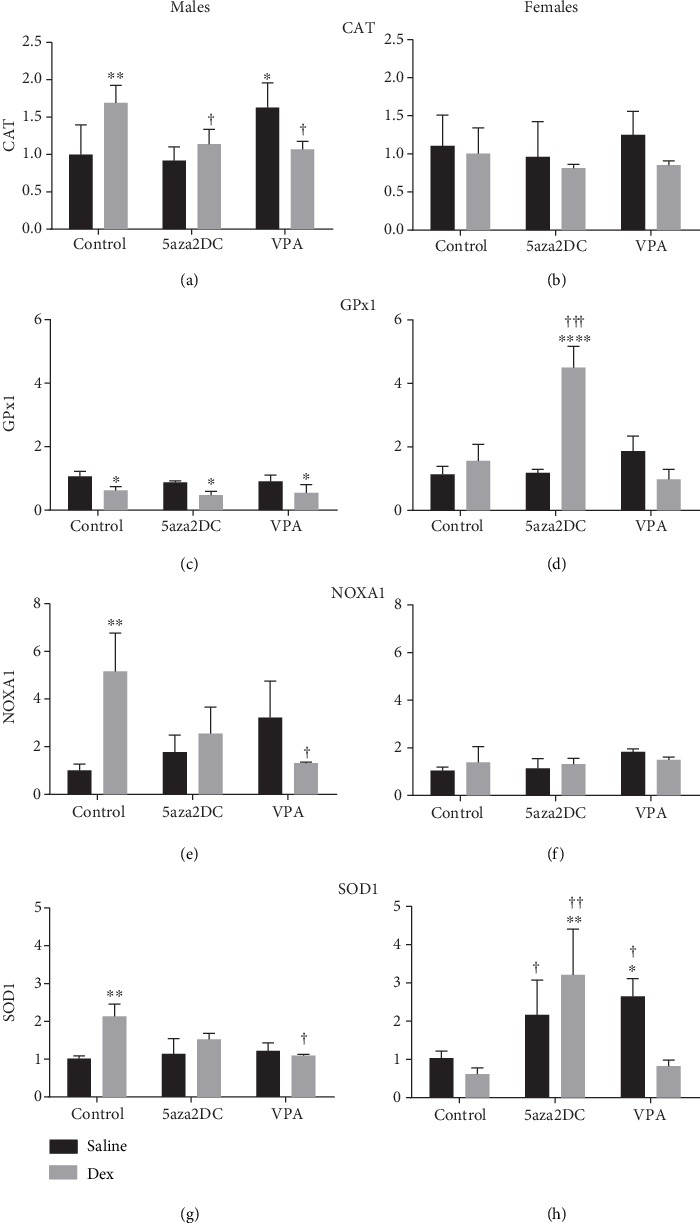
Antioxidant and ROS-related gene expression results from the RT^2^ profiler array (Qiagen). Quantification of qPCR gene expression which showed significance from the RT^2^ profiler array including (a) catalase (CAT), (b) glutathione peroxidase 1 (GPx1), (c) NADPH oxidase activator 1 (Noxa1), and (d) superoxide dismutase 1 (SOD1). Two-way ANOVA (Fisher LSD test): statistical significance is represented as ^∗/†^*P* ≤ 0.05, ^∗∗/††^*P* ≤ 0.01, ^∗∗∗/†††^*P* ≤ 0.001, and ^∗∗∗∗/††††^*P* ≤ 0.0001. Saline-Control and Dex-Control are shown here for reference, and results have been described previously. Data are presented as mean ± SEM. ∗ indicates significance relative to Saline-Control, and † indicates significance relative to Dex-Control. *N* = 3‐5 per group.

The results from the RT^2^ profiler array suggest distinct differences in oxidative stress markers between Control-Dex and Control-Saline groups predominantly in male offspring. Prenatal Dex exposure increased the expression of catalase (CAT) (1.7-fold), Noxa1 (5.2-fold), and SOD1 (2.1-fold) in males and not females (Figures 9(a)–9(h)).

5aza2DC was effective in reducing altered ROS-related genes in Dex-exposed offspring as CAT (1.1-fold), Noxa1 (1.7-fold), and SOD1 (1.5-fold) to expression levels comparable to Saline-Control (Figures 9(a), 9(e), and 9(g)), with the exception of GPx1 which remained unchanged (Figure 9(c)). Programmed females in the 5aza2DC-Dex group display different trends than males. No change was seen with CAT and Noxa1; however, GPx1 (4.5-fold) and SOD1 (3.2-fold) were increased compared to Saline-Control and Dex-Control (Figures 9(d) and 9(h)). SOD1 was also significantly increased in unprogrammed 5aza2DC-Saline females (2.2-fold) compared to Dex-Control but not significantly different then Saline-Control (Figure 9(h)).

VPA was effective in reducing altered expression of CAT (1.1), Noxa1 (1.3), and SOD1 (1.1) in Dex-exposed offspring (Figures 9(a), 9(e), and 9(g)). Again, GPx1 expression was not recovered in this group with VPA (0.5) and remained reduced compared to Saline-Control (Figure 9(c)). In unprogrammed males that received VPA injections, a significant increase in CAT expression (1.6) was found as compared to Saline-Control (Figure 9(a)). VPA-Saline males show a similar trend for increased Noxa1 expression (3.2); however, the results did not reach significance (Figure 9(e)).

Programmed females that received VPA injections did not display significant changes in the expression of CAT, GPx1, or Noxa1 compared to the Dex-programmed group (Figures 9(b), 9(d), and 9(f)). Interestingly, unprogrammed females that received VPA displayed elevated SOD1 expression levels (2.6-fold) compared to Saline-Control and Dex-Control (0.6-fold) (Figures 9(h)).

**Figure 10:**
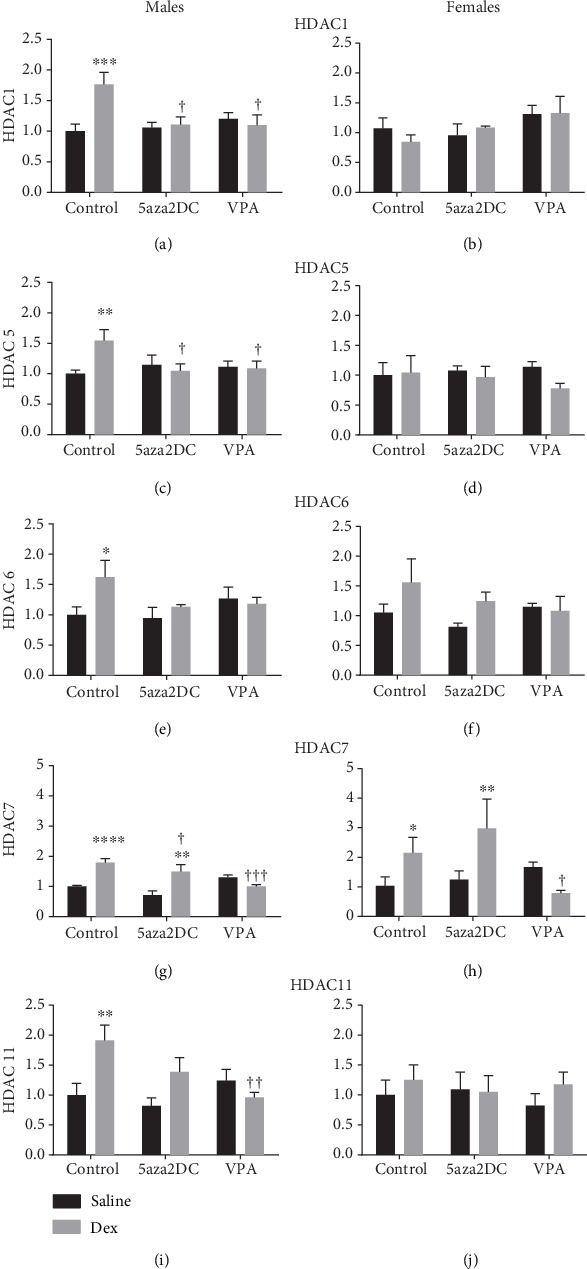
HDAC gene expression results from the RT^2^ profiler array (Qiagen). (a) Histone deacetylase (HDAC) 1, (b) HDAC5, (c) HDAC6, (d) HDAC7, and (e) HDAC11 for males and females. Two-way ANOVA (Fisher LSD test): statistical significance is represented as ^∗/†^*P* ≤ 0.05, ^∗∗/††^*P* ≤ 0.01, ^∗∗∗/†††^*P* ≤ 0.001, and ^∗∗∗∗/††††^*P* ≤ 0.0001. Saline-Control and Dex-Control are shown here for reference, and results have been described previously. Data are presented as mean ± SEM. ∗ indicates significance relative to Saline-Control, and † indicates significance relative to Dex-Control. *N* = 3‐5 per group.

Dex exposure resulted in significant changes in HDAC expression levels in male offspring including HDAC1 (1.8-fold), HDAC5 (1.5-fold), HDAC6 (1.6-fold), HDAC7 (1.8-fold), and HDAC11 (1.9-fold) (Figures 10(a), 10(c), 10(e), 10(g), and 10(i)). Dex-programmed females displayed similar trends as males in HDAC6 and HDAC7, with HDAC7 (2.1-fold) reaching significance (Figures 10(f) and 10(h)).

Interestingly, 5aza2DC was effective in reducing Dex-mediated altered HDAC expression (Figures 10(a)–10(j)) with the exception of HDAC7 for males and females (Figures 10(g) and 10(h)).

Similar to 5aza2DC, Dex-programmed male offspring that received VPA injections displays significantly reduced expression of HDAC1, HDAC5, and HDAC11 and also reducing HDAC7 compared to Dex-Control (Figures 10(a), 10(c), 10(e), 10(g), and 10(i)). Females also show a similar trend as males as both HDAC6 and HDAC7 were reduced to control (Figures 10(f) and 10(h)). VPA-Saline males did not show any significant changes in HDAC gene expression compared to Saline-Control animals.

## 4. Discussion

Epigenetic inhibition through DNMTi via 5aza2DC or through HDACi via VPA attenuated the GC-mediated fetal programming (FP) of the HPA axis particularly in male offspring. Male offspring showed a significant reduction in majority of the CA enzyme and transcription factor mRNA gene expression, a reduction in PNMT protein levels, and a reduction in circulating Epi and BP. Females also display similar trends in response to VPA; however, 5aza2DC actually increased expression of CA enzymes and increased plasma Epi, suggesting sex-specific responses to epigenetic inhibitors; however, BP remained at control levels, indicating it was effective in attenuating hypertension to some extent, potentially through alternate mechanisms.

### 4.1. Epigenetic Inhibitors Attenuate GC-Mediated Fetal Programming of Blood Pressure and Impact on Body Weight

There is evidence that epigenetics is implicated in hypertension and is suspected to be involved in GC-mediated FP of hypertension. Epigenetic inhibitors were employed to investigate the link between epigenetics and GC-mediated programming of the HPA. Histone acetylation generally promotes euchromatin conformation promoting gene transcription by allowing access to the gene for transcription machinery. As a result, histone deacetylation via HDACs canonically leads to heterochromatin formation and gene silencing; however, HDACs have also been shown to promote gene expression in some cases [19, 20]. VPA mediates HDACi and thus gene activation by reducing protein activity, potentially via binding to the catalytic centre [21]. VPA exhibits HDACi across several classes of HDACs including class 1 and some class 2 HDACs [1–5, 7] and is a more potent inhibitor of class IIb HDACs [6, 10] [22]. VPA also displays some activity outside of HDACi, and HDACs can deacetylate proteins other than histones to alter activity.

DNMTi via 5aza2DC is a result of the cytidine analogue replacing native cytosine residues during replication. DNMT then binds these altered bases resulting in covalent bond, decreasing soluble DNMT concentrations [23]. Furthermore, there is evidence that azacitidines increase proteasomal degradation of DNMTs, as a result multiple mechanisms are likely involved [23, 24]. The reduction in soluble DNMT is thought to lead to hypomethylation and gene activation. Consequently, DNMTi should promote gene activation, although this is not always the case. There is evidence that 5aza2DC can induce chromatin remodeling and promote gene activation by altering histone methylation status [25]. Antithetically, DNMTi may also reduce gene expression when reduced promoter methylation allows increased repressor protein binding [26].

Previous studies have shown that GC exposure during the third trimester of pregnancy leads to the development of hypertension in adulthood [1, 2]. Indicative of *in utero* Dex exposure, programmed animals display decreased birth weight [27]. In our current study, as expected, Dex programming has reduced male and female offspring body weight up until week 11, before epigenetic inhibitor administration (Figures 2(a) and 2(b)). Animals at week 14 did not display significant differences in body weight; however, this is likely not due to epigenetic inhibitor administration as treatment groups remain similar to Control-Dex animals (Figure 2(c)). However, interestingly, epigenetic inhibition successfully attenuated GC-mediated changes in BP. Following epigenetic inhibitor administration at the end of week 14, both 5aza2DC and VPA reduced BP down to control levels in males and females (Figures 3(a)–3(d)), attenuating the hypertensive phenotype and confirming a role for epigenetics in GC-programmed hypertension. Previous evidence has highlighted a role for epigenetics in the development of hypertension in SHR [9]. Additionally, evidence has linked prenatal GC exposure to altered methylation that is tissue specific in guinea pigs [3]. This study describes a novel method of attenuating postnatal increases in BP as a result of GC-mediated FP in rats via HDACi or DNMTi.

### 4.2. The Role of Epigenetics in CA Biosynthesis in GC-Programmed Offspring (mRNA and Protein)

Methylation of CA enzyme promoter regions is suspected to alter gene expression and binding of transcription factors. In a human cell line, TH has been shown to contain a CpG site which can be methylated, altering Sp1 binding, and DNMTi via 5aza2DC increased TH expression [28]. DBH promoter methylation has also been suggested to modulate expression and affect behaviour [29]. PNMT may be regulated by promoter methylation; however, this has yet to be investigated.

Interestingly, inhibition of methylation via 5aza2DC or HDACi via VPA significantly attenuated the majority of programmed elevations in CA enzymes in males including TH, DBH, PNMT, SP1, EGR1, and GR (Figures 4(a), 4(c), 4(e), 5(a), 5(c), and 5(e)). In females, DNMTi did not attenuate CA enzyme programming; however, HDACi was effective (Figures 5(b), 5(d), and 5(f)). Interestingly, in both sexes, the administration of VPA without prior prenatal Dex exposure significantly increased CA enzyme expression, in many instances above that of Dex alone (Figures 4(a)–4(f)). HDAC6 has been shown to bind GR forming a repressor complex which has the potential to inhibit CA enzyme expression as they are regulated by GR [30]. As a result, inhibition of HDACs via VPA may serve to increase active GR by preventing formation of the repressor complex, although this remains unknown. Furthermore, Dex has been shown to increase this association between HDAC6 and GR increasing repression of GR target genes; however, the impact of Dex on this association has not been studied from a FP perspective [30]. HDACs also catalyze deacetylation of proteins outside of histones and can contribute significantly to cell signalling mechanisms [31]. GR is one such protein and can be deacetylated directly by HDACs (1, 2, and 3), altering how GR impacts transcription [10, 32]. Studies in a rat model of Cushing's syndrome show HDACi abolished hypertension potentially via inhibition of GR activity by increasing its acetylation directly [10]. As a result, it is difficult to determine whether HDACi impacts CA enzymes and transcription factor expression, or transcription factor binding activity through direct association and repression, or by catalyzing acetylation and activity. Elucidating the mechanisms leading to altered CA enzyme expression is difficult; however, evidence suggests a potential role for Dex-HDAC-GR interactions.

Interestingly, changes in CA enzyme expression due to either epigenetic inhibitor only resulted in reduced PNMT and GR protein levels as TH and DBH remained elevated (Figures 6(a)–6(f)). Altered expression of miRNA may explain differences in gene and protein levels; indeed, miRNA have been shown to alter CA synthesis and secretion, and additional regulatory mechanisms are likely involved [33]. miRNA have also been shown to regulate corticosteroidogenesis [34] and dopamine synthesis [35] and transport [36] TH [37], SP1 [33], and GR levels [38].

Importantly, the reduction in PNMT protein resulted in reduced plasma Epi levels for males given VPA or 5aza2DC. VPA also mirrored these results in females. Interestingly, 5aza2DC increased plasma Epi for Dex-exposed females. This was unexpected as PNMT mRNA, and overall, BP is comparable to control animals. The protective role of estrogen in GC-mediated FP is complex. Estrogen has been shown to alter the expression of epigenetic regulators including HDACs and DNMTs, and the presence of ER on a gene promoter has been correlated with its methylation status [39]. Estrogen has also been implicated in complex cycling of gene methylation/demethylation via DNMTs and inhibition of this cycling resulted in transcriptional activation [40]. This type of complex transcriptional regulation may account for estrogen's protective effects on programming. Furthermore, if the protective effects of estrogen, specifically with respect to Epi synthesis or degradation, involve DNMT-mediated changes in gene expression, it is plausible that this may explain the effects of 5aza2DC on Epi in Dex programming. Furthermore, estrogen plays a protective role on the cardiovascular system via its ability to reduce ROS production and increase antioxidant enzyme levels [41]. ROS levels impact gene expression and have been shown to alter the expression of PNMT [42].

Dex exposure results in reduced NE levels (Figures 8(a) and 8(b)), potentially a result of increased PNMT to mediate conversion of NE to Epi. When given 5aza2DC, both sexes display increased NE above that of control, indicating a potential build-up of substrate for Epi synthesis. Conversely, VPA decreased NE levels, comparable to control in males, and NE remained reduced and comparable to Dex alone in females (Figure 8(b)). Again, it is not surprising that NE levels differ between sexes in Dex-exposed animal given VPA, since as mentioned previously, estrogen can alter HDAC expression among others [39].

### 4.3. The Relationship between ROS and Epigenetics in the Fetal Programing of Hypertension

Increased GCs drive increased metabolic activity and thus mitochondrial ROS production[43], and these changes in ROS may mediate FP via GC; however, the mechanism remains undetermined. Evidence for both ROS and epigenetic dysregulation has been described in hypertensive models [3, 7, 9, 44, 45]. There is significant overlap between ROS and epigenetic gene regulation in hypertensive programming as either pathway can alter the other. For example, ROS produced via NADPH oxidases (Nox) increase DNMT and HDAC activity, which in turn can silence or upregulate Nox depending on the protein [46]. HDACi has proven to reduce Nox expression and ROS production in a rat model of pulmonary hypertension [47]. Expression of antioxidant enzymes also impact the expression of epigenetic machinery. Increased expression of SOD1 and GPx1 alters histone acetylation and methylation differentially; however, if it occurs through histone acetyltransferase or HDAC or another mechanism, regulation is unknown [26]. This study shows that Dex exposure resulted in significant changes in antioxidant and prooxidant enzyme expression as well as altered HDAC expression. Interestingly, 5aza2DC attenuated dysregulation of antioxidant/oxidant enzyme expression in males including CAT, Noxa1, and SOD1 but not GPx1 (Figure 9), indicating that altered expression of these enzymes due to GC programming involves DNMTs. Interestingly, females which generally do not display dysregulation of ROS pathways in response to Dex show that 5aza2DC in combination with Dex exposure significantly increases antioxidant enzymes Gpx1 and SOD1 (Figures 9(b) and 9(d)). Taken together, these results indicate a potential link between antioxidant response to Dex exposure and DNMTs, with sex-specific responses. It is possible that elevated SOD1 and reduced GPx1 act together in association with increased Noxa1 in Dex-programmed males to drive altered HDAC expression as seen (Figures 10(a), 10(c), 10(e), 10(g), and 10(i)) and thus impacting gene expression within the CA biosynthesis pathway. How DNMTs are implicated in this pathway and whether ROS is upstream of epigenetic regulation remains to be determined. Inhibition of either epigenetic machinery or ROS has proven effective in remediating programming effects in previous studies. HDACi via VPA has been shown to decrease ROS and the Nox catalytic subunit gp91phox in SHR [9] and to reduce programmed ROS production due to low protein maternal diet [48]. Furthermore, maternal antioxidant administration has been shown to prevent GC-related programming in mice [45].

## 5. Conclusion

The fetal programming of hypertension via GCs involves both ROS and epigenetics in the pathology of adult disease. Specifically, GC FP results in dysregulation of ROS through altered expression of CAT, Gpx1, SOD1, and Noxa1 and implicating epigenetic regulators including HDACs and DNMTs. HDACi and DNMTi are effective in attenuating HPA axis programming of CA biosynthetic enzymes including PNMT, the production of CAs including Epi, and attenuating the hypertensive phenotype particularly in male offspring. It is clear that both epigenetics and ROS are connected and drive expression of each other. This feed forward loop appears to be implicated in the pathogenesis of Dex-mediated hypertension. When combined with previous studies, targeting this mechanism at the level of ROS or epigenetics are effective in remediating altered expression of key regulators in both pathways and preventing or attenuating GC-mediated FP of hypertension.

## Figures and Tables

**Table 1:** Primer specification and qPCR parameters.

Gene target	Primer sequences	Amplicon size (bp)	Amplification conditions	cDNA input (ng)	Primer input (nM)
PAH NM_012619.2	**F:**GCTGCTAAGCTAGACACCTCA **R**:CTTGTTTCCTGCCCAAAGTCT	105	1. 95°C, 2 min 2. 95°C, 1 min 3. 60°C, 1 min 4. 72°C, 1 min 5. Plate read 6. Go to line 2, 39 more times 7. Melting curve from 55-95°C, read every 1°C, hold 10 sec	7.5	600

TH L22651	**F:**GCGACAGAGTCTCATCGAGGAT **R:**AGAGCAGGTTGAGAACAGCATT	150	1. 95°C, 2 min 2. 95°C, 1 min 3. 58°C, 1 min 4. 72°C, 1 min 5. Plate read 6. Go to line 2, 29 more times 7. Melting curve from 55-95°C, read every 1°C, hold 10 sec	7.5	600

DBH NM_013158	**F:**TTCCCCATGTTCAACGGACC **R:**GCTGTGTAGTGTAGACGGATGC	240	1. 95°C, 2 min 2. 95°C, 1 min 3. 58°C, 1 min 4. 72°C, 1 min 5. Plate read 6. Go to line 2, 29 more times 7. Melting curve from 55-95°C, read every 1°C, hold 10 sec	7.5	600

PNMT X75333	**F:**CATCGAGGACAAGGGAGAGTC **R:**GCAGCGTCGTGATATGATAC	219	1. 95°C, 2 min 2. 95°C, 1 min 3. 60°C, 1 min 4. 72°C, 1 min 5. Plate read 6. Go to line 2, 39 more times 7. Melting curve from 55-95°C, read every 1°C, hold 10 sec	7.5	300

SP1 D12768.1	**F:**CAGACTAGCAGCAGCAATACCA **R:**TGAAGGCCAAGTTGAGCTCCAT	224	1. 95°C, 2 min 2. 95°C, 1 min 3. 58°C, 1 min 4. 72°C, 1 min 5. Plate read 6. Go to line 2, 29 more times 7. Melting curve from 55-95°C, read every 1°C, hold 10 sec	7.5	600

EGR1 AY551092.1	**F:**TTTCCACAACAACAGGGAGAC **R:**CTCAACAGGGCAAGCATACG	261	1. 95°C, 2 min 2. 95°C, 1 min 3. 58°C, 1 min 4. 72°C, 1 min 5. Plate read 6. Go to line 2, 29 more times 7. Melting curve from 55-95°C, read every 1°C, hold 10 sec	7.5	600

GR NM_012576.2	**F:**TGCTGGAGGTGATTGAACCC **R:**TCACTTGACGCCCACCTAAC	111	1. 95°C, 2 min 2. 95°C, 1 min 3. 58°C, 1 min 4. 72°C, 1 min 5. Plate read 6. Go to line 2, 29 more times 7. Melting curve from 55-95°C, read every 1°C, hold 10 sec	7.5	600

Beta actin NM_031144	**F:**TCTGTGTGGATTGGTGGCTCT **R:**GACTCATCGTACTCCTGCTTG	83	1. 95°C, 2 min 2. 95°C, 1 min 3. 58°C, 1 min 4. 72°C, 1 min 5. Plate read 6. Go to line 2, 29 more times 7. Melting curve from 55-95°C, read every 1°C, hold 10 sec	7.5	600

RPL29 NM_017150	**F:**TGAGAGGTAGGGTCCCGTTT **R:**TCAGTTCTGGGACCTGACCA	144	1. 95°C, 2 min 2. 95°C, 1 min 3. 58°C, 1 min 4. 72°C, 1 min 5. Plate read 6. Go to line 2, 29 more times 7. Melting curve from 55-95°C, read every 1°C, hold 10 sec	7.5	600

## Data Availability

All relevant data are within the manuscript files.
